# Treatment for locally resectable stage IIIC1r cervical cancer: surgery or chemoradiotherapy?

**DOI:** 10.1186/s12885-024-11944-0

**Published:** 2024-02-15

**Authors:** Mei-ling Zhong, Yin-chuan Liu, Jian-tong Yang, Ya-nan Wang, Mei-hong Ao, Yun Xiao, Si-yuan Zeng, Mei-rong Liang

**Affiliations:** 1 Department of Gynecological Oncology, Jiangxi Maternal and Child Health Care Hospital, 330008 Nanchang, Jiangxi China; 2 Department of Radiation Oncology, Jiangxi Cancer Hospital of Nanchang Medical College, 330029 Nanchang, Jiangxi China; 3 Graduate School, Medical College of Nanchang University, No. 318, Bayi Avenue, 330006 Nanchang, Jiangxi China

**Keywords:** Cervical cancer, Pelvic lymph node metastasis, Radical hysterectomy, Chemoradiotherapy

## Abstract

**Objective:**

The aim of this study was to compare the therapeutic value and treatment-related complications of radical hysterectomy with those of concurrent chemoradiotherapy (CCRT) for locally resectable (T1a2–T2a1) stage IIIC1r cervical cancer.

**Methods:**

A total of 213 patients with locally resectable stage IIIC1r cervical cancer who had been treated at Jiangxi Maternal and Child Health Care Hospital between January 2013 and December 2021 were included in the study and classified into two groups: surgery (148 patients) and CCRT (65 patients). The disease-free survival (DFS) rate, overall survival (OS) rate, side effects, and economic costs associated with the two groups were compared.

**Results:**

43.9% (65/148) patients in the surgical group had no pelvic lymph node metastasis, and 21of them did not require supplementary treatment after surgery due to a low risk of postoperative pathology. The median follow-up time was 46 months (range: 7–108 months). The five-year DFS and OS rates of the surgery group were slightly higher than those of the CCRT group (80.7% vs. 75.1% and 81.6% vs. 80.6%, respectively; *p* > 0.05). The incidences of grade III–IV gastrointestinal reactions in the surgery and CCRT groups were 5.5% and 9.2%, respectively (*p* = 0.332). Grade III–IV myelosuppression was identified in 27.6% of the surgery group and 26.2% of the CCRT group (*p* = 0.836). The per capita treatment cost was higher for the surgery group than for the CCRT group (RMB 123, 918.6 0 vs. RMB 101, 880.90, *p* = 0.001).

**Conclusion:**

The therapeutic effects and treatment-related complications of hysterectomy and CCRT are equivalent in patients with locally resectable stage IIIC1r cervical cancer, but surgery can provide accurate lymph node information and benefit patients with unnecessary radiation.

## Introduction

Cervical cancer is a leading health problem for women worldwide. In 2020, about 604,000 new cases were diagnosed, and 342,000 people died of the disease [1]. Lymph node (LN) metastasis is an independent factor for the prognosis of cervical cancer and plays a crucial role in the 2018 International Federation of Gynecological Organizations (FIGO) staging system [2]. Specifically, when imaging reveals the presence of metastasis in the pelvic lymph nodes, the cervical cancer is subclassified as stage IIIC1r; paraaortic LN metastasis is classified as stage IIIC2r. As reported, different local tumor stages are closely associated with the prognosis of patients with Stage IIIc1r [3–4]. The National Comprehensive Cancer Network guidelines recommend concurrent chemoradiotherapy (CCRT) and brachytherapy for all stage IIIC1r patients (category 1), but there is no hierarchical therapy for those patients [5]. In addition, diagnostic imaging tools, such as CT, MRI, and PET-CT, are associated with varying rates of false negatives and positives [6–7]. Therefore these uncertainties may lead to some over treatment or insufficient cure problems in imaging-based concurrent CCRT.

Prior to the publication of the 2018 FIGO staging system for cervical cancer, radical hysterectomy was recommended for stage Ia2–IIa1 patients, regardless of the presence or absence of LN metastases, in China and Japan [8–9]. Some studies have shown that the oncological outcomes of surgery ± CCRT are not inferior to those of direct CCRT for locally resectable stage IIIC1r patients [10–11]. However, there is a lack of high-quality prospective randomized clinical trials and clear evidence for the treatment of locally resectable stage IIIC1r cervical cancer. Therefore, we conducted a retrospective study to compare the therapeutic value and treatment-related complications of radical hysterectomy with those of concurrent chemoradiotherapy (CCRT) for locally resectable (T1a2–T2a1) stage IIIC1r cervical cancer.

## Materials and methods

### Patients

The medical records of patients with locally resectable (T1a2–T2a1) stage IIIC1r cervical cancer who underwent initial treatment at Jiangxi Maternal and Child Health Hospital between January 2013 and December 2021 were retrospectively reviewed. The inclusion criteria were as follows: (1) age: less than 70 years; (2) stage (FIGO 2018): IIIC1r but with a locally resectable (T1a2–T2a1) tumor; (3) histologic subtypes: squamous cell carcinoma, adenocarcinoma, or adenosquamous carcinoma; (4) no contraindications for surgery or chemoradiotherapy; and (5) complete follow-up data. None of the patients had previously received abdominal radiotherapy, and patients with distant metastasis confirmed by imaging were ruled out.

### Imaging data

MRI and/or CT scans were used to determine the presence of pelvic LN metastasis, and the data were reviewed by at least two radiologists. A short axis diameter greater than 10 mm and the presence of central necrosis in the lymph node were the criteria used to determine pelvic LN metastasis via MRI or CT. Central necrosis was defined as a central density of less than 20 H in unenhanced and enhanced CT images or an intranodal isointense (to water) area in T1- and T2-weighted MRIs, with no enhancement after contrast material administration [12–13]. Patients whose MRI or CT scans indicated the presence of pelvic LN metastasis were classified as having stage IIIC1r cervical cancer.

### Treatment

The patients in the surgery group underwent radical hysterectomy, and observations or supplementary treatments were performed after surgery based on the postoperative risk factors. Following primary hysterectomy, observation is recommended for patients with stage IA2, IB or IIA1 disease who have negative nodes, negative parametria, negative margins, and no other cervical risk factors (Sedlis Criteria). Pelvic EBRT with (or without) concurrent platinum-containing chemotherapy is administrated for patients with stage IA2, IB, or IIA1 disease who have negative lymph nodes but have large primary tumors, deep stromal invasion, and/or LVSI. Postoperative CCRT with (or without) vaginal brachytherapy is recommended for patients with positive pelvic nodes, positive surgical margin, and/or positive parametrium; A dose of 45 to 50 Gy in standard fraction with intensity-modulated radiation therapy (IMRT) is generally given for postoperative EBRT.

For patients in the CCRT group, pelvic EBRT to a total dose of 45–50 Gy was carried out. An additional 30–40 Gy of high-dose brachytherapy was administered to the primary cervical tumor in fractions of 5–7 Gy. The total point A dose was 80 Gy for small-volume cervical tumors and ≥ 85 Gy for large-volume cervical tumors. During external beam radiotherapy, weekly platinum-based combination chemotherapy was administered. All patients signed an informed consent form prior to treatment. This study was approved by the Ethics Committee of Jiangxi Maternal and Child Health Hospital.

### Follow-up

After completing treatment, the patients were reexamined once every 3 months in the first year, once every 3–6 months in the second year, once every 6–12 months in the third to fifth years, and once every 12 months after five years. Patients with high-risk disease were evaluated more frequently. Local recurrence and distant metastasis were recorded upon performing follow-up imaging or pathological studies. The primary objective of this study was to determine the disease-free survival (DFS) and overall survival (OS) rates of the patients in each group. Late treatment-related adverse events were evaluated according to the Common Terminology Criteria for Adverse Events Version 4.0 [14].

### Statistical methods

Two independent samples t-tests were used for intergroup comparisons of the measurement data, while the chi-square test or Fisher’s exact test was used for the comparison of count data. A Kaplan–Meier survival analysis was performed to calculate the survival rates, and a log-rank test was used for survival rate comparison and stratified analysis. A Cox proportional hazards model was used for the multivariate analysis of prognostic factors. *P* values < 0.05 were statistically significant.

## Results

A total of 213 eligible patients were included in this study: 148 patients in the surgical group and 65 patients in the CCRT group. 43.9% (65/148) patients in the surgical group had no pelvic lymph node metastasis, and 21 of them did not require supplementary treatment after surgery due to a low risk of postoperative pathology. The median age at diagnosis was 49 years (range: 24–69 years). The characteristics of the two patient groups are listed in Table 1.

**Table 1:** Comparison of participants’ characteristics

Characteristics	Surgery	Chemoradiotherapy	*p* Value
	n(%)	n(%)	
Age			
<50y	90(60.8)	20(30.8)	0.000
≥ 50y	58(39.2)	45(69.2)	
Tumor subtype			
Squamous	124(83.8)	63(97.0)	0.012
Adenocarcinoma	19(12.8)	1(1.5)	
Aenosquamous	5(3.4)	1(1.5)	
T staging			
T1b1	121(81.8)	41(63.1)	0.003
T2a1	27(18.2)	24(36.9)	
Differentiation degree			
Medium - high	137(92.6)	59(90.8)	0.656
Low	11(7.4)	6(9.2)	
Tumor size			
≤ 2 cm	60(40.5)	23(35.4)	0.477
>2 cm, ≤4 cm	88(59.5)	42(64.6)	
Number(PLNM)			
<2	66(44.6)	32(49.2)	0.532
≥ 2	82(55.4)	33(50.8)	
Size(PLNM)			
>10 mm, ≤ 15 mm	119(80.4)	49(75.4)	0.408
>15 mm	29(19.6)	16(24.6)	

Abbreviations: PLNM = Pelvic lymph nodes metastasis

### Treatment outcomes and survival rates

**Fig. 1:**
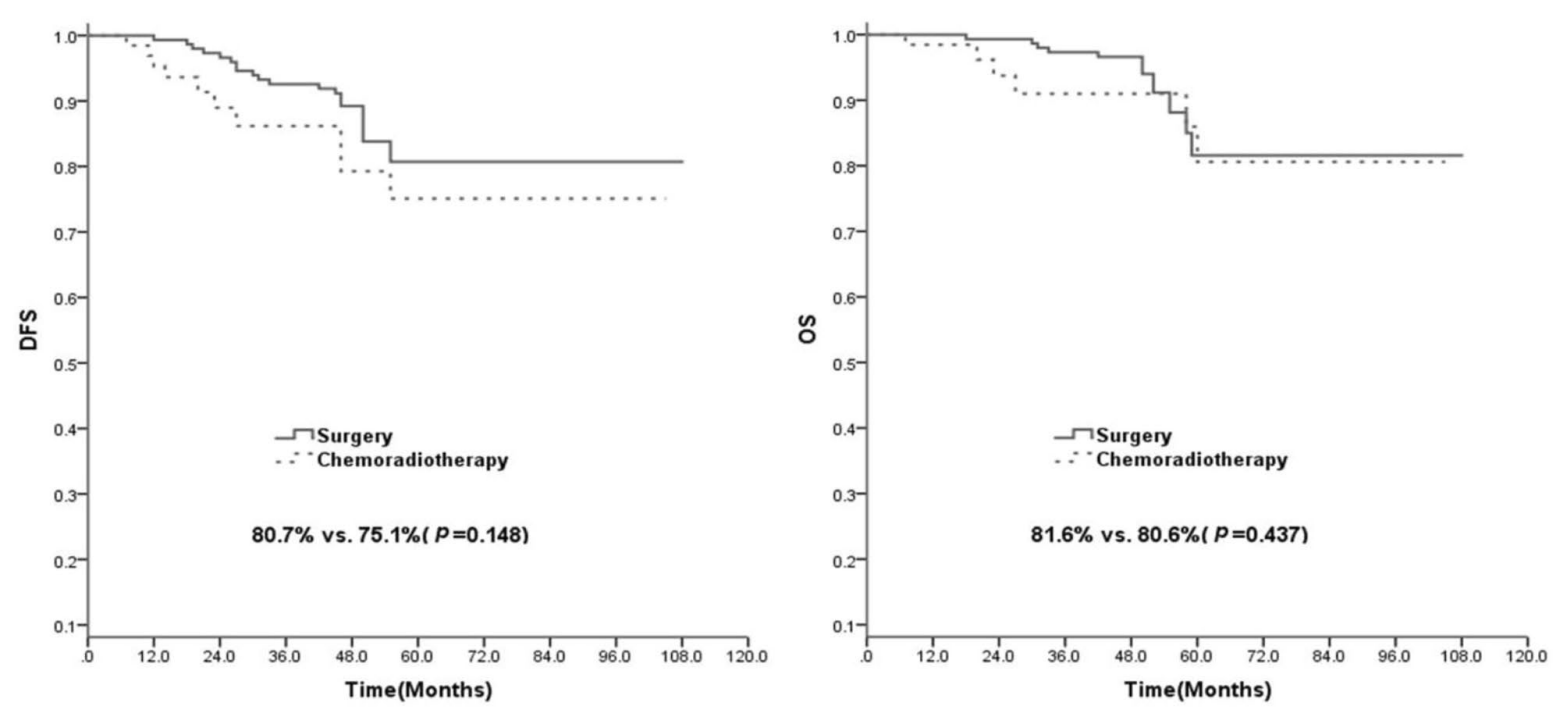
Disease-free survival (DFS) and overall survival (OS) rates of patients with locally resectable stage IIIC1r cervical cancer after surgery (*n* = 148) and after concomitant chemoradiotherapy (CCRT, *n* = 65)

The median follow-up time was 46 months (range: 7–108 months). A total of 28 patients experienced recurrence, and 16 patients died during the follow-up period. The five-year DFS rates were 80.7% for the surgery group and 75.1% for the CCRT group; the difference between the two groups was not statistically significant (*p* = 0.148). The five-year OS rate was slightly higher for the surgery group than for the CCRT group (81.6% vs. 80.6%, *p* = 0.437; Fig. 1).

### Prognostic factor analysis

A univariate analysis revealed that the local tumor T stage and the number and size of imaging-positive pelvic lymph nodes were associated with the patients’ five-year DFS (all *p* < 0.05). The number of imaging-positive pelvic LNs was also associated with the five-year OS (*p* = 0.038). A multivariate analysis showed that the local tumor T stage and the number and size of imaging-positive pelvic LNs were independent risk factors for the five-year DFS. The number of imaging-positive pelvic LNs was also an independent risk factor for the patients’ five year-OS. These results are presented in Table 2.

**Table 2:** Analysis of the prognostic factors affecting DFS and OS

	Univariate analysis				Multivariate analysis			
Variables	DFS(%)	*p* value	OS(%)	*p* value	DFS		OS	
					Wals χ^2^*p* value		Wals χ^2^*p* value	
**Age**		0.115		0.162	0.377	0.539	0.647	0.421
<50y	86.1		86.5					
≥ 50y	72.7		76.8					
**Treatment**		0.148		0.437	1.303	0.254	0.425	0.514
Surgery	80.7		81.6					
Chemoradiotherapy	75.1		80.6					
**Tumor subtype**		0.210		0.312	0.024	0.988	0.002	0.999
Squamous	77.5		79.7					
Adenocarcinoma	100		100					
Aenosquamous	80.0		100					
**T staging**		**0.017**		0.104	3.861	**0.049**	1.204	0.273
T1b1	84.6		87.8					
T2a1	65.9		68.4					
**Differentiation degree**		0.713		0.615	0.012	0.912	0.109	0.742
Medium - high	80.9		81.3					
Low	70.3		87.8					
**Tumor size**		0.228		0.311	0.195	0.658	0.613	0.434
≤ 2 cm	84.8		85.2					
>2 cm, ≤4 cm	75.8		79.4					
**Number(PLNM)**		**0.019**		**0.038**	4.068	**0.044**	4.013	**0.045**
<2	89.7		90.8					
≥ 2	52.1		55.6					
**Size(PLNM)**		**0**		0.164	7.118	**0.008**	0.675	0.411
>10 mm, ≤ 15 mm	84.8		84.7					
>15 mm	56.7		68.7					

Abbreviations: PLNM = Pelvic lymph nodes metastasis

### Treatment-related side effects

Among the patients in the surgery group, 21 did not require adjuvant treatment after surgery due to a low risk of postoperative pathology; therefore, only 127 patients received CCRT after surgery. Grade III–IV myelosuppression was identified in 27.6% (35/127) of the patients in the surgery group and 26.2% (17/65) of those in the CCRT group (*p* = 0.836). The incidences of grade III–IV gastrointestinal reactions were 5.5% (7/127) and 9.2% (6/65) in the surgery and CCRT groups, respectively. The difference between the two groups was not statistically significant (*p* = 0.332). The results are shown in Table 3.

**Table 3:** Treatment related complications in surgery and chemoradiotherapy group

	Surgery N(%)	Chemoradiotherapy N(%)	χ²	*p* Value
gastrointestinal reactions				
I-II	120(94.5)	59(90.8)		
III-IV	7(5.5)	6(9.2)	0.942	0.332
myelosuppression				
I-II	92(72.4)	48(73.8)		
III-IV	35(27.6)	17(26.2)	0.043	0.836

### Comparison of treatment costs

The average treatment cost was 123,918.6 yuan for the patients in the surgery group and 101,880.9 yuan for those in the CCRT group. An independent samples t-test was performed to compare the treatment costs of the two groups. The results revealed that the difference between the average treatment costs of the two groups was statistically significant (t = 4.309, *p* = 0.001).

## Discussion

Since its publication, the 2018 FIGO staging system for cervical cancer has received a great amount of research attention in China and other countries [15–17]. Stage IIIC1r in this system includes patients with pelvic lymph node metastasis (excluding paraaortic LN metastasis and distant metastasis) indicated by imaging, regardless of tumor size or degree of parametrial invasion. There is currently no recommended treatment for patients with stage IIIC1r cervical cancer worldwide, especially for patients with locally resectable (T1a2–T2a1) tumors.

The present study showed that the therapeutic efficacies of surgery and CCRT in treating locally resectable stage IIIC1r cervical cancer patients are equivalent. This finding is consistent with that of the ABRAX study, which involved a retrospective analysis of 515 cervical cancer patients with lymph node involvement detected intraoperatively; 361 patients completed the planned radical surgery, and 154 did not [18]. After a median follow-up of 58 months, no survival benefit associated with the completion of radical hysterectomy was found, regardless of tumor size or histological type. In Kashima et al.’s study [19], the recurrence-free survival and overall survival rates of the surgery group were inferior to those of the CCRT group (*p* = 0.02 and 0.04, respectively). However, only 31 locally resectable (T2) stage IIIC1r patients enrolled in this study, and 16 patients in the surgery group received adjuvant chemotherapy instead of radiotherapy, which may have contributed to the group’s inferior clinical outcome [19].

However, in this study, 43.9% (65/148) patients of surgical group had no pelvic lymph node metastasis, and 21 of them did not require supplementary treatment after surgery due to a low risk of postoperative pathology. That means the false positive of FIGO 2018 staging was 43.9%, and indicated that if the CT or MRI suggestion of IIIc1r, almost half of cases would benefit from surgery with unnecessary radiation. MRI is a standard pre-treatment imaging modality for cervical cancer, while the accuracy of MRI for detection of nodal metastasis is not so well. The reported positive predictive value were 20–66% for CT scan, and 0–27% for MRI scan. The negative predictive values of the imaging techniques were 53–92% for CT scan, an 75–91% for MRI [20]. PET- CT performs better than CT and MRI while there is still 10% false- negative rate [6–7, 20]. Therefore these uncertainties may lead to some over treatment or insufficient cure problems in imaging-based concurrent CCRT. And surgery can provide accurate lymph node information and benefit patients with unnecessary radiation.

Combined treatment is presumably associated with increased morbidity. However, in the present study, there were no significant differences between the two groups in terms of the grade III–IV myelosuppression incidence rates and grade III–IV gastrointestinal reactions. This was consistent with literature reports [19, 21–22]. Notably, treatment cost was higher for the surgery group than for the CCRT group (*p* = 0.001).

Since stage IIIC1r is diagnosed by imaging, the accuracy of imaging plays a crucial role in staging. Regardless of whether MRI or PET-CT is used, false positives are inevitable. Surgery can help clarify the status of a patient’s lymph nodes and thus guide subsequent treatment. In our study, 65 patients in the surgery group were confirmed to have negative pathology, and 21 of them did not require supplementary treatment after surgery due to a low risk of postoperative pathology. This indicates that surgery not only reduces the tumor burden but also allows for accurate evaluations of imaging-positive lymph nodes. Nevertheless, CCRT is also a feasible treatment option, as it allows for avoiding the dual impact of surgical risk and the side effects of radiation and chemotherapy on patients when its efficacy is equivalent to that of surgical treatment, thus improving patients’ quality of life and reducing their financial burdens. Before lymph node factors were included in the cervical cancer staging system, surgery was often the preferred treatment for patients with early-stage cervical cancer (stage IA–IIA). In China and most economically underdeveloped regions, surgery is typically considered the best way to achieve a radical cure. However, when selecting treatment options for patients with locally resectable stage IIIC1r cervical cancer who strongly demand surgical treatment, it is vital to fully inform them of the efficacy levels, side effects, and costs of surgery and concurrent chemotherapy so that they can make informed, reasonable choices.

As this study was a retrospective analysis, it had certain limitations that must be acknowledged. First, the comparative analysis of patient clinicopathological data revealed age differences between the surgery and CCRT groups, with a greater proportion of young patients in the surgery group. However, in a previous study [23], the prognosis of cervical cancer patients was not related to age. Second, the local tumor status differed between the two groups, with a higher proportion of T1b1 local tumors in the surgery group than in the CCRT group. Third, the sample size was too small to draw strong conclusions.

## Conclusion

The therapeutic effects and treatment-related complications associated with surgery and CCRT for locally resectable stage IIIC1r cervical cancer patients are equivalent, but surgery can provide accurate lymph node information and benefit patients with unnecessary radiation. The international literature on the prognosis of patients with stage IIIC1r cervical cancer mostly includes retrospective analyses. High-quality prospective, multicenter, large-sample, randomized controlled studies should be conducted in the future to confirm the results of the present study and to provide better hierarchical management strategies for locally resectable stage IIIC1r cervical cancer.

## Data Availability

All data generated or analyzed during this study are included in this article. Further enquiries can be directed to the corresponding author.
